# Deletion of Superoxide Dismutase 1 Blunted Inflammatory Aortic Remodeling in Hypertensive Mice under Angiotensin II Infusion

**DOI:** 10.3390/antiox10030471

**Published:** 2021-03-16

**Authors:** Yasunaga Shiraishi, Norio Ishigami, Takehiko Kujiraoka, Atsushi Sato, Masanori Fujita, Yasuo Ido, Takeshi Adachi

**Affiliations:** 1 Division of Environmental Medicine, National Defense Medical College Research Institute, 3-2 Namiki, Tokorozawa Saitama 359-8513, Japan; fujitama@ndmc.ac.jp; 2 Department of Internal Medicine, Division of Cardiovascular Medicine, National Defense Medical College, 3-2 Namiki, Tokorozawa Saitama 359-8513, Japan; ishigaminoriohome@yahoo.co.jp (N.I.); nbqnx536@ybb.ne.jp (T.K.); atsushi19821005@yahoo.co.jp (A.S.); yido@bu.edu (Y.I.); tadachi@ndmc.ac.jp (T.A.)

**Keywords:** superoxide dismutase 1, angiotensin II, vascular hypertrophy, inflammation, hydrogen peroxide, interleukin 6, STAT3

## Abstract

Superoxide dismutase (SOD) is an enzyme that catalyzes the dismutation of two superoxide anions (O_2_^·−^) into hydrogen peroxide (H_2_O_2_) and oxygen (O_2_) and is generally known to protect against oxidative stress. Angiotensin II (AngII) causes vascular hypertrophic remodeling which is associated with H_2_O_2_ generation. The aim of this study is to investigate the role of cytosolic SOD (SOD1) in AngII-induced vascular hypertrophy. We employed C57/BL6 mice (WT) and SOD1 deficient mice (SOD1^−/−^) with the same background. They received a continuous infusion of saline or AngII (3.2 mg/kg/day) for seven days. The blood pressures were equally elevated at 1.5 times with AngII, however, vascular hypertrophy was blunted in SOD1^−/−^ mice compared to WT mice (WT mice 91.9 ± 1.13 µm versus SOD1^−/−^ mice 68.4 ± 1.41 µm *p* < 0.001). The elevation of aortic interleukin 6 (IL-6) and phosphorylation of pro-inflammatory STAT3 due to AngII were also blunted in SOD1^−/−^ mice’s aortas. In cultured rat vascular smooth muscle cells (VSMCs), reducing expression of SOD1 with siRNA decreased AngII induced IL-6 release as well as phosphorylation of STAT3. Pre-incubation with polyethylene glycol (PEG)-catalase also attenuated phosphorylation of STAT3 due to AngII. These results indicate that SOD1 in VSMCs plays a role in vascular hypertrophy due to increased inflammation caused by AngII, probably via the production of cytosolic H_2_O_2_.

## 1. Introduction

In our bodies, the reactive oxygen species (ROSs) are constantly produced, and they are high reactivity which potentially damages the biological systems. However, an appropriate amount of ROSs is necessary for biological processes including cell signaling [1,2] and defense system. In normal condition, the proper balance of pro-oxidant-antioxidant system maintains biological homeostasis [3]. Perturbing this balance leads to oxidative stress and causes many disorders including vascular diseases.

Angiotensin II (AngII) is the established pathophysiology of hypertension and vascular hypertrophy. Part of this pathophysiology is caused by ROSs produced by NADPH oxidase (NOX) [4,5]. NOX is activated by AngII through protein kinase C (short term), Rac [6] and c-Src-dependent pathways which stimulate p47phox phosphorylation and translocation [7]. NOX family enzymes (NOX 1,2,4) increase superoxide anion (O_2_^·−^) or hydrogen peroxide (H_2_O_2_) levels in the vascular smooth muscle cell (VSMC) [8] immediately after AngII binding (~1 min), which last for 24 h [9,10]. Some NOXs localize at specific subcellular compartments such as caveolae and endosomes [11] and the produced O_2_^·−^ moves through anion chloride channel (CLC-3) [12] and H_2_O_2_ diffuses through aquaporin channels, which changes local oxidation state.

O_2_^·−^ is slowly dismutated to H_2_O_2_ spontaneously (rate constant = 8 × 10^4^/mol/s). In the presence of nitric oxide (NO^−^), O_2_^−^ quickly reacts with NO^−^ to form nitrogen peroxide acid (OONO^−^) (rate constant = 4–16 × 10^9^/mol/s). NO· functions as a vasodilator and vascular protector, however, OONO^−^ does not induce vasodilation and further causes organ damage because of its strong reactivity. Superoxide dismutase (SOD) is one of the antioxidant systems and could rapidly catalyze two O_2_^·−^ into H_2_O_2_ and O_2_ (rate constant = 2 × 10^9^/mol/s). The resulting H_2_O_2_ serves as a substrate for catalases, which ultimately break it down into harmless H_2_O and O_2_ [13]. Therefore, elimination of O_2_^·−^ by SOD protects from producing OONO^−^ and is likely to relax vascular tension which is related to blood pressure [14] and protect vascular damage. On the other hand, H_2_O_2_ produced by SOD plays a role in signal pathways activated with AngII, which leads to protein and DNA synthesis in VSMCs in vitro [15,16] and excessive production of H_2_O_2_ causes aortic VSMC hypertrophy [17,18]. AngII induces ROSs production and SOD would change the ratio of O_2_^·−^ and H_2_O_2_ local concentration. In other words, SOD helps to relax vascular tension and protect from vascular damage by eliminating O_2_^·−^; however, the H_2_O_2_ produced by SOD would therefore be an important mediator for AngII-induced vascular hypertrophy. SOD may have both positive and negative effects on vascular pathophysiology.

SOD is a metal-containing enzyme, and the catalytic reaction is performed by cyclic oxidation and reduction of metal ions. There are three main types of SOD: copper/zinc-containing SOD (CuZnSOD), iron-containing SOD (FeSOD), and manganese-containing SOD (MnSOD). SOD genes have been relatively preserved during evolution [13], but their localization and types have changed. As for CuZnSOD, it is found in the periplasmic space in Gram-negative bacteria, but in animals, it is present as a dimer in the cells (SOD1) and in the nucleus, mitochondrial intermembrane space and peroxisomes in addition to the cytoplasm [19]. Extracellular CuZnSOD (SOD3) is present in a tetrameric state extracellularly attached to heparan sulfate proteoglycans. SOD1 and SOD3 are regulated by separate genes; SOD3 is related to fungal CuZnSOD and may be a more classical form than SOD1.

The SOD of primitive organisms was FeSOD, but it is thought to have fallen out of use with the rise in atmospheric O_2_, and now FeSOD is absent in animals and fungi, and only present in chloroplasts in plants. Instead, MnSOD was used during evolution, and FeSOD and MnSOD have similar structures [19]. In plants and animals, MnSOD (SOD2) is present in the mitochondrial matrix in a tetrameric state.

Because of the cytosolic location, SOD1 could be important to cell biology. We aimed to investigate the effects of SOD1 in vascular pathophysiology, which changed the local oxidate state in vivo. To date, some studies reported the role of SOD1 in blood pressure and vascular pathophysiology, however, the result was inconsistent and controversial [20,21,22,23,24]. The involvement of local H_2_O_2_ in AngII-induced aortic hypertrophy has not yet been completely elucidated in vivo.

In the present in vivo study, we hypothesized that SOD1 exerts its effects on vascular pathology by altering the local oxidative state in vivo and aimed to investigate its effects. We employed SOD1 deficient mice (SOD1^−/−^) and compared them with wild-type mice (WT). Without any intervention, there was no difference in blood pressure and aortic pathology. By infusing a relatively high dose of AngII (3.2 mg/kg/day) for 1 week, although the response to AngII-induced hypertension was comparable, the aortic hypertrophy was blunted in SOD1^−/−^ mice compared with WT mice.

## 2. Materials and Methods

### 2.1. Experimental Animals

Male SOD1^−/−^ mice were obtained from Jackson Laboratory (Bar Harbor, Maine). These mice were backcrossed to C57BL/6J strain mice for 8 generations as we did previously [25]. Next, we interbred heterozygous SOD1^+/−^ mice to obtain wild-type (WT) mice and homozygous SOD1^−/−^ mice within the same litter. Animals were maintained in a temperature-controlled facility on a 12 h light/dark cycle (lights on from 7:00 a.m. to 7:00 p.m.) and fed freely with a normal chow diet (CLEA Japan, Inc., Higashiyama, Meguro-ku, Tokyo) and water. The genotype of each mouse was checked by polymerase chain reaction on DNA isolated from a tail or ear biopsy samples using the following primers:

WT 5′-TGAACCAGTTGTGTTGTCAGG-3′
WT 5′- TCCATCACTGGTCACTAGCC-3′
Mutant 5′- TGTTCTCCTCTTCCTCATCTCC-3′
Mutant 5′- ACCCTTTCCAAATCCTCAGC-3′

All experimental protocols were approved by National Defense Medical College Board for Studies in Experimental Animals (approval number: 10063).

### 2.2. AngII Infusion

We implanted 12- to 16-week-old male mice with osmotic mini-pumps (Alzet model 1007D; DURECT Corp, Cupertino, CA) containing AngII (A9525 Sigma-Aldrich Corp, St. Louis, MO, USA) or saline (*n* = 22~26 per group). The delivery rate of AngII was 3.2 mg/kg/day for seven days as in the study of Wang HD et al. [26]. All surgical procedures in this experiment were performed by aseptic manipulation, and disinfectant solution (10% povidone-iodine solution, Meiji Co., Ltd., Tokyo, Japan) was used. Mice were sedated in an anesthesia box filled with 3.5% sevoflurane. Then, under continuous inhalation of 2.5% to 3.5% sevoflurane, we made a mid-scapular skin incision with surgical scissors and fine forceps to make a small pocket and placed an osmotic mini-pump inside. The wound was sutured with silk thread. 5 mg/kg of meloxicam was injected subcutaneously as an analgesic.

### 2.3. VSMC Cell Culture

We obtained 6-week-old male Wistar rats from CLEA Japan, Inc. (Tokyo, Japan). All surgical procedures in this experiment were performed by aseptic manipulation, and disinfectant solution (10% povidone-iodine solution, Meiji Co., Ltd., Tokyo, Japan) was used. Rats were sedated in an anesthesia box filled with 5% sevoflurane. After sufficiently deep anesthesia was obtained, the rats were sacrificed to death by cervical dislocation. Saline reflux was performed from the heart, and the thoracic aorta was removed. After cleaning up the fat tissue around the aorta, the aorta was cut longitudinally and opened. Then the media was peeled off from the adventitia. The isolated media was cut into small pieces and incubated with 0.75 mg/mL collagenase and 0.125 mg/mL elastase for 30 min to 60 min by pipetting to disperse the cells. The collected cells were grown in 1.0 g/L glucose Dulbecco’s modified Eagle’s medium (#11885 Life Technologies Japan Ltd. Tokyo, Japan) with 10% FBS and penicillin/streptomycin. To decrease expression of SOD1, we incubated VSMCs with SOD1 siRNA (#1330003 (set of 3 Oligos; Oligo IDs: RSS303072, RSS303073, RSS303074) Invitrogen) and Lipofectamine RNAiMAX (Invitrogen) for 72 h, and after changing the medium to 0.1% FBS and leaving for 24–48 h, performed experiments with or without AngII or Interleukin-6 (IL-6). To inhibit SOD1 activity pharmacologically, we used DETC (diethyldithiocarbamate) which was a copper-chelating compound. 0.1 mM DETC was added to VSMC culture fluid 4 h before the addition of AngII. Catalase was administered 24 h before AngII administration. We used polyethylene glycol-conjugated catalase (PEG-catalase) (C4963 Sigma-Aldrich Corp, St. Louis, MO. USA). It is expected to be highly stable while maintaining good reactivity by binding polyethylene glycol to catalase [27].

### 2.4. Measurement of SOD Activity

SOD activities in red blood cells were determined using SOD Assay Kit (#43379000 Wako Pure Chemical Industries, Ltd. Osaka, Japan) following the kit’s instructions. The erythrocytes were washed three times with 0.9% NaCl. The washed erythrocytes (0.1 mL) were lysed using distilled water (0.5 mL) and mixed. To remove the hemoglobin, 1.2 mL of an ethanol/chloroform (1:1 *v/v*) mixture was added to an aliquot (0.6 mL) of hemolysate. After centrifugation for 10 min at 1600× *g*, the supernatant was collected for SOD assay. This kit uses the nitro blue tetrazolium (NBT) method to measure SOD activity. O_2_^·−^ produced by the addition of xanthine oxidase (XO) reduces NBT to form nitroblue tetrazolium diformazan, which shows absorbance at 560 nm and the absorbance was measured by the plate reader (iMark™ Microplate Absorbance Reader, BIO-RAD, Hercules, CA, USA). In the presence of SOD, some of O_2_^·−^ is dismutated to H_2_O_2_ and O_2_, inhibiting the formation of diformazan. The SOD activity in a sample is measured by its inhibition rate as shown in Equation (1) [28].

SOD activity (Inhibition rate %) = ((Abs _BL_ − Abs _BL__BL_) − (Abs _S_ − Abs _SBL_))/(Abs _BL_ − Abs _BL__BL_)∗100 (1)

Abs _BL_: Absorbance of the blind test (water)
Abs _BLBL_: Absorbance of reagent blind test (water without XO)
Abs _S_: Absorbance of sample test
Abs _SBL_: Absorbance of sample blind test (sample without XO)

### 2.5. Measurement of Blood Pressure

Systolic blood pressure was measured by the tail-cuff method (MK-2000; Muromachi Kikai Co., Ltd. Tokyo, Japan) without anesthesia between 6:00 a.m. and 8:00 a.m., following the instructions as previous reports [20,22,26]. To reduce the stress associated with the BP measurements, mice were introduced into the plastic restrainer and measured BP from one week before the main experiment and 10 times averages were calculated at each determination.

### 2.6. Tissue Preparation and Histology

The mice were deeply anesthetized and perfused with 0.9% saline from the left ventricular apex, followed by perfusion fixation with 4% paraformaldehyde. Aortas were fixed in 10% formalin for 48 h. The 2 mm thoracic aorta rings were cut off at 4 mm from the aortic arch and embedded in paraffin. These were sectioned (4-µm thickness) and stained with hematoxylin and eosin (HE). For immunohistochemistry, SOD1 was visualized with anti-human SOD1 polyclonal antibodies (Enzo Life Sciences, Inc., Farmingdale, NY, USA) using a Vectastain Elite ABC kit (Vector Laboratories, Burlingame, CA, USA) with diamino-benzidine as the substrate, following the instructions.

### 2.7. Measurement of Aorta Medial Area

We cut out the aorta ring 5 mm from the aortic arch and measured the thoracic aorta medial area using a HE Stain; Elastica van Gieson (EVG) stain, and Masson’s trichrome (Masson’s) stained cross-section slides. The media was defined as the area between the internal and external elastic lamina. For each mouse, the average of ten consecutive cross-sections was measured and quantified using the NIH Image software (National Institutes of Health, public domain software, Bethesda, MD, USA).

### 2.8. Measurement of Extracellular and Intracellular H_2_O_2_ Levels

We measured the serum derivatives of reactive oxygen metabolites (d-ROMs) using FREE carpe diem (Diacron International s.r.l. Grosseto, Italy) as an extracellular H_2_O_2_ indicator. The serum d-ROMs test detects hydro-peroxide (R–OOH) and measurements are expressed as Carratelli units (CARR U), with 1 CARR U corresponding to 0.08 mg/dL H_2_O_2_. Oxidized glutathione (GSSG) levels and reduced glutathione (GSH) levels were measured in red blood cells as an intracellular H_2_O_2_ indicator using GSH/GSSG Kit (Oxis International Inc., Foster City, CA, USA), following the instructions.

Superoxide productions in aortic tissue were visualized by dihydroxyethidine (DHE) staining of aortic frozen samples. Vascular GSH/GSSG ratios were assessed with CoulArray^®^ detector (#5600A, ESA Inc., Chelmsford, MA, USA) after TCA extraction [29].

### 2.9. Western Blotting

Aortas were homogenized with a glass-glass homogenizer on ice and dissolved in homogenization buffer (Tris-HCl 20 mmol/L, pH 7.4, NaCl 150 mmol/L, Na_2_EDTA 1 mmol/L, EGTA 1 mmol/L, 1% NP-40, sodium pyrophosphate 2.5 mmol/L, beta-glycerophosphate 1 mmol/L, Na_2_VO_4_ 1 mmol/L) supplemented with PMSF 1 mmol/L and protease inhibitor cocktail (Sigma-Aldrich Corp, St. Louis, MO, USA). VSMCs were dissolved in homogenization buffer and collected with a scraper. After brief sonication (10 s, ×3, on ice), these samples were centrifuged at 10,000× *g* for 10 min at 4 °C. Protein concentrations were assessed by the Bradford assay with BSA as a standard. Aortic and VSMCs lysates were resolved by SDS-PAGE and transferred to PVDF membranes (30 V, 135 min). The membranes were incubated with the following antibodies overnight at 1:1000 dilution: SOD1 (Enzo Life Sciences, Inc., Farmingdale, NY, USA), SOD2 (Assay Designs, Inc., Ann Arbor, MI, USA), Phospho-STAT3 (Cell Signaling Technologies, Danvers, MA, USA), STAT3 (Cell Signaling Technologies, Danvers, MA, USA), Phospho-ERK1/2 (Cell Signaling Technologies, Danvers, MA, USA), ERK1/2 (Cell Signaling Technologies, Danvers, MA, USA), Phospho-Akt (Cell Signaling Technologies, Danvers, MA, USA), Akt (Cell Signaling Technologies, Danvers, MA, USA) and GAPDH (Cell Signaling Technologies, Danvers, MA, USA). They were then incubated with peroxidase-conjugated anti-mouse or anti-rabbit antibodies (Cell Signaling Technologies, Danvers, MA, USA) overnight. The specific protein amount was detected after immersing in Enhanced Chemiluminescence (ECL) (Thermo Fisher Scientific Inc., Waltham, MA, USA) and light emission was detected and captured by a LAS-3000 Imager (Fujifilm, Tokyo, Japan).

### 2.10. Quantitative Real-Time PCR

Total RNA was isolated from the aortas using TRI reagent (Sigma-Aldrich Corp, St. Louis, MO, USA). cDNA was synthesized using SuperScript III reverse transcriptase (Thermo Fisher Scientific Inc., Waltham, MA, USA) according to the manufacturer’s protocol. mRNA expression was assessed quantitatively by real-time PCR with Power SYBR Green PCR Master Mix (Thermo Fisher Scientific Inc., Waltham, MA, USA). Samples were run in duplicate on the ABI PRISM 7700 (Thermo Fisher Scientific Inc., Waltham, MA, USA). The following oligonucleotide primer pairs were used (Table 1).

## 3. Results

### 3.1. Baseline Characteristics and Blood Pressure

**Figure 1:**
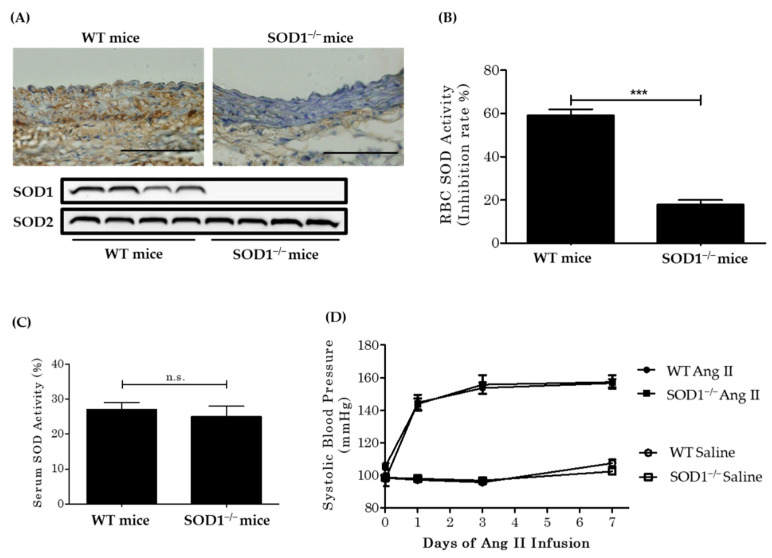
The efficiency of superoxide dismutase (SOD1) deletion and effect of AngII infusion on systolic blood pressure. (**A**) Immunohistochemistry (upper part) and Western blot analysis (lower part) of aortas from WT mice and SOD1^−/−^ mice using anti-SOD1 antibodies. (Scale bars = 100 µm.) SOD activity in red blood cells (**B**) and serum (**C**). Red blood cells (RBCs) do not have mitochondria so SOD activity would be that of cytosol SOD (SOD1). (*n* = 22~26 per group. *** *p* < 0.001 n.s.: not significant). (**D**) Saline or AngII (3.2 mg/kg per day) was chronically infused using osmotic mini-pumps. Systolic blood pressure was measured by the tail-cuff method (*n* = 12 per group). Error bars represent SEM. WT: wild type; SOD1^−/−^: superoxide dismutase 1 deficient; AngII: angiotensin II.

We confirmed that SOD1 was deficient in aortas from SOD1^−/−^ mice by immunohistochemistry and western blot analysis as previously reported [25]. SOD2 expression was comparable in the aorta of WT and SOD1^−/−^ mice (Figure 1A). SOD activity in red blood cells was markedly decreased in SOD1^−/−^ mice (WT mice 59.1 ± 2.9 versus SOD1^−/−^ mice 17.9 ± 2.0 (*n* = 22–26)) (Figure 1B), although plasma SOD activity was similar. (Figure 1C). SOD activity remained in SOD1^−/−^ erythrocytes. This may be a result of the spontaneous dismutation of O_2_^·−^ or the residual SOD3 activity of the samples. The baseline systolic blood pressure was similar between groups (WT mice 98.3 ± 2.0 mmHg (*n* = 12) versus SOD1^−/−^ mice 98.5 ± 2.1 mmHg (*n* = 12)). AngII infusion increased systolic blood pressure similarly and there was no significant difference in blood pressure between the two strains (WT mice 156.6 ± 2.5 mmHg (*n* = 12) versus SOD1^−/−^ mice 157.3 ± 4.2 mmHg (*n* = 12) at 7 days) (Figure 1D).

### 3.2. Histological and Morphometric Analysis

**Figure 2:**
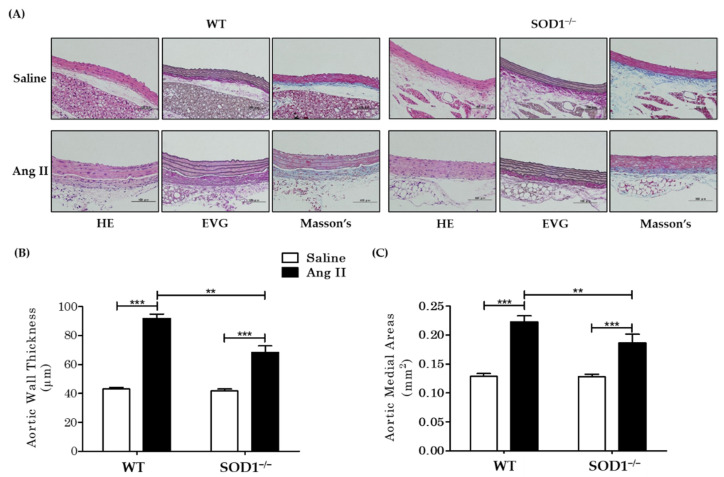
Microscopic appearance of aortas and quantitative analysis. (**A**) Hematoxylin–eosin (HE), Elastica van Gieson (EVG), and Masson’s staining of aortas. WT mice with saline (upper left), SOD1^−/−^ mice with saline (upper right), WT mice with AngII (lower left), SOD1^−/−^ mice with AngII (lower right). (Scale bar = 100 μm.) (**B**) Quantification of aorta wall thickness and (**C**) medial areas measured by NIH Image software (*n* = 10–12 per group. ** *p* < 0.01 *** *p* < 0.001) Error bars represent SEM. HE: Hematoxylin–Eosin Stain; EVG: Elastica van Gieson stain; Masson’s: Masson’s trichrome stain.

The collected aortas were subjected to HE staining, EVG staining, and Masson’s trichrome staining (Figure 2A) (Supplemental Figure S1). In the saline-treated group, no changes were observed in SOD1^−/−^ mice compared to WT mice, while AngII-treated mice showed hypertrophy of vascular smooth muscle cells with associated thickening of the tunica media and thickening of the adventitia due to fibrosis. Nuclei were enlarged, nuclear staining by hematoxylin was weakened, and cytoplasmic vacuolation was observed, suggesting that cell death occurred. The above findings with AngII administration were more pronounced in WT but were attenuated in SOD1^−/−^. Quantitative analysis of aortic vessel wall thickness showed that Ang II-induced aortic wall thickening, which was observed in WT, was attenuated in SOD1^−/−^ (Figure 2B,) (WT mice 91.9 ± 1.13 µm versus SOD1^−/−^ mice 68.4 ± 1.41 µm *p* < 0.001). Vascular tunica media area was also weakened in SOD1^−/−^ (WT mice 0.223 ± 0.0051 mm^2^ versus SOD1^−/−^ mice 0.186 ± 0.004 mm^2^ *p* = 0.001) (Figure 2C).

### 3.3. Extracellular and Intracellular H_2_O_2_ Levels In Vivo

**Figure 3:**
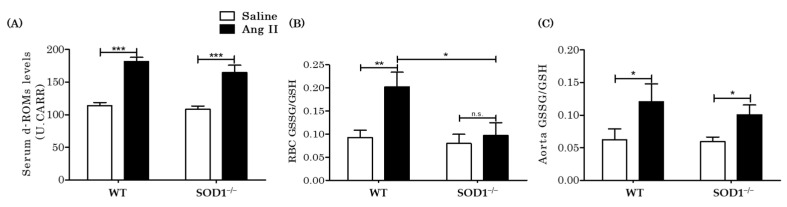
Extracellular and intracellular H_2_O_2_ levels in blood and aortas in vivo. (**A**) Derivatives of reactive oxygen metabolites (d-ROMs), which detect hydro-peroxide (R–OOH), were measured as an extracellular H_2_O_2_ indicator (*n* = 12 per group. *** *p* < 0.001). (**B**) Oxidized glutathione (GSSG)/reduced glutathione (GSH) ratios were measured in RBCs as an indicator of intracellular H_2_O_2_ (*n* = 8 per group. * *p* < 0.05 ** *p* < 0.01 n.s.: not significant). (**C**) GSSG/GSH ratios in aortas (*n* = 10~11 per group. * *p* < 0.05). Error bars represent SEM. ROS: reactive oxygen species; WT: wild type; SOD1^−/−^: superoxide dismutase 1 deficient; Ang II: angiotensin II; d-ROMs: derivatives of reactive oxygen metabolites; RBC: red blood cell; GSSG: oxidized glutathione; GSH: reduced glutathione; DHE: Dihydroethidium.

H_2_O_2_ is produced from O_2_^·−^ by SOD and we hypothesized that the intracellular H_2_O_2_ would be specifically low in SOD1^−/−^ mice because SOD1 is localized in the cytosol and the mitochondrial intermembrane space. We measured both the extracellular and intracellular H_2_O_2_ levels in WT and SOD1^−/−^ mice. We used serum d-ROMs levels as an indicator of extracellular H_2_O_2_ levels [30]. The serum d-ROMs test detects hydro-peroxide products (R–OOH), which reflect serum H_2_O_2_ levels [31]. The serum d-ROMs levels were elevated similarly in both types of mice under AngII infusion (WT mice from 113.7 ± 5.2 to 181.5 ± 6.5 U.CARR versus SOD1^−/−^ mice from 108.7 ± 4.8 to 164.7 ± 11.3 U.CARR (*n* = 12)) (Figure 3A).

Next, we measured the oxidized glutathione (GSSG) levels and reduced glutathione (GSH) levels in red blood cells as an indicator of cytosolic H_2_O_2_ levels. The H_2_O_2_ in the cytosol is promptly metabolized to H_2_O by catalase, glutathione peroxidase (Gpx), and peroxiredoxins [32]. When Gpx metabolizes H_2_O_2_, it oxidizes GSH to GSSG. An increase in GSSG/GSH ratio thus reflects an increase in intracellular H_2_O_2_ level. GSSG/GSH ratios in red blood cells were increased in WT mice by AngII, while in SOD1^−/−^ mice, the ratios did not increase (WT mice from 0.093 ± 0.016 to 0.20 ± 0.032 versus SOD1^−/−^ mice from 0.080 ± 0.020 to 0.097 ± 0.027 (*n* = 8)) (Figure 3B). The GSSG/GSH ratio in the aorta was also assessed: the GSSG/GSH ratio increased with AngII and tended to increase less in SOD1^−/−^ but did not differ significantly (WT mice from 0.062 ± 0.017 to 0.12 ± 0.027 versus SOD1^−/−^ mice from 0.059 ± 0.007 to 0.10 ± 0.015 (*n* = 10–11)) (Figure 3C). We also assessed aortic O_2_^·−^ levels with the DHE stain. The DHE intensities were significantly increased by AngII in both WT and SOD1^−/−^ mice, and there was no difference between both mice (Supplemental Figure S2). These results were not consistent with that for red blood cells measured above. These discrepancies were thought to be due to the fact that the O_2_^−^ produced by Ang II is catalyzed by SOD2, which is mainly localized in the mitochondrial matrix. In the SOD1^−/−^ mice, SOD2 was still functioning and would hinder detection of SOD1 activity in aortas. As there are no mitochondria in red blood cells, SOD1 activity would have become apparent.

### 3.4. Western Blot Analysis for Downstream Signaling of AngII in Aortas In Vivo

**Figure 4:**
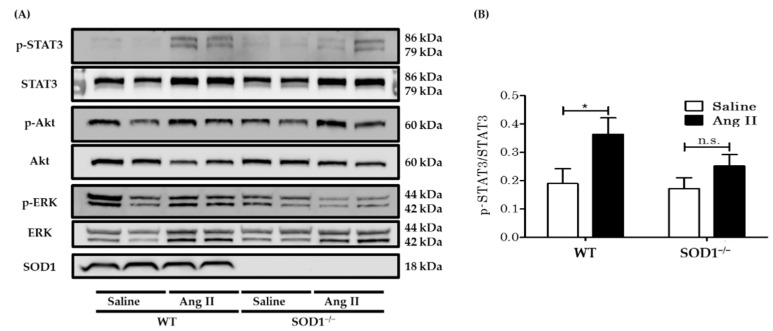
The protein expressions of aortas from WT and SOD1^−/−^ mice with saline or AngII. (**A**) Representative results of Western blot analyses of phosphorylated STAT3, phosphorylated Akt, phosphorylated ERK1/2, and SOD1 in aortas from WT and SOD1^−/−^ mice, with saline or AngII. (**B**) Quantitative analyses by densitometry of immunoblots expressed as ratios of phosphorylated STAT3 to STAT3. (*n* = 8 per group. * *p* < 0.05 n.s.: not significant) Error bars represent SEM. STAT3: signal transducer and activator of transcription 3; ERK: extracellular signal-regulated kinase.

Various studies have indicated that AngII stimulates a series of protein kinases, including ERK1/2, Akt/protein kinase B, and JAK2/STAT3 pathways, leading to cell proliferation in vitro [33,34,35,36,37]. We investigated the expression and phosphorylation of ERK1/2, Akt, and STAT3 in proteins from the aortas in vivo. Unlike previous in vitro studies [34,36,37], the phosphorylation of ERK1/2 and Akt was not augmented by seven days of AngII-infusion in both groups (Figure 4A). On the other hand, AngII-infusion enhanced phosphorylation of STAT3 in the aortas from WT mice, which was blunted in those from SOD1^−/−^ mice (Figure 4A,B). We, therefore, considered phosphorylation of STAT3 to be a mechanism for AngII-induced cell modification in vivo.

### 3.5. Serum Inflammatory Cytokines Levels and Aortic mRNA Expressions In Vivo

**Figure 5:**
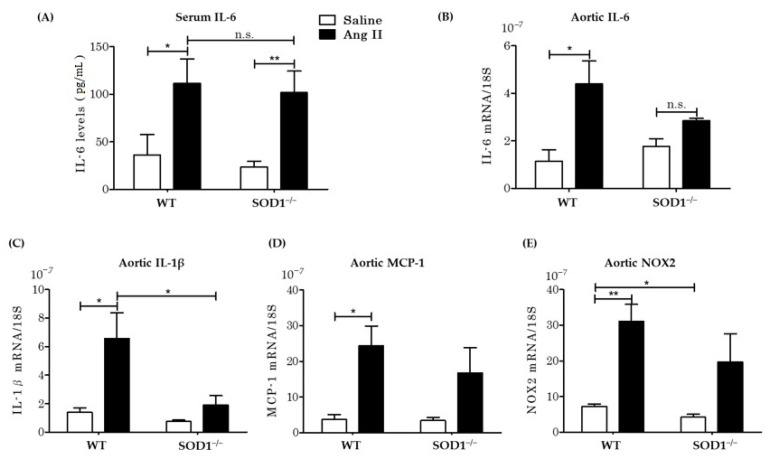
Serum IL-6 concentration and mRNA expression in aortas. (**A**) Serum IL-6 levels were analyzed by the ELISA test (*n* = 9–10 per group. * *p* < 0.05 ** *p* < 0.01 n.s.: not significant). (**B**–**E**) The local aortic IL-6 (**B**), IL-1β (**C**), MCP-1 (**D**) and NOX2 (**E**) levels were detected as mRNA expressions, which were compensated with an internal control: 18S (*n* = 4–8 per group; * *p* < 0.05, ** *p* < 0.01 n.s.: not significant). Error bars represent SEM. IL-6: interleukin 6; IL-1β: interleukin 1β; MCP-1: Monocyte Chemotactic Protein-1; NOX2: NADPH Oxidase 2.

STAT3 is phosphorylated by cytokines and IL-6 is representative of cytokines that phosphorylate STAT3. Previous studies [38,39,40] have shown that Ang II induces IL-6 synthesis in VSMCs and that the reaction requires ROSs from the NOX system [38]. This suggests that IL-6 secreted by the vessel wall may be responsible for the difference in aortic wall changes observed after Ang II administration as described above. Serum IL-6 levels were elevated similarly in both types of mice by AngII infusion (Figure 5A). To investigate aortic local IL-6 levels, we assessed mRNA expression in the aortas from the four groups. AngII upregulated IL-6 mRNA expression in the aorta of WT mice but suppressed the increase in expression in SOD1^−/−^ mice (Figure 5B). We also examined other inflammatory cytokines. AngII induced expression of IL-1β and MCP-1 mRNA in aortas from WT mice, which was blunted in aortas from SOD1^−/−^ mice (Figure 5C,D). On the other hand, there were no significant differences in serum TNF-α levels and the expression of TNF-α mRNA in aortas in any of the four experimental groups (Supplemental Figure S3A,B). These data indicated that AngII induced inflammations, however, the local inflammation in the aortas were blunted in SOD1^−/−^ mice.

As AngII activates NOX family enzymes (NOX 1,2,4) [9,10] in order to produce ROSs, we also assessed the mRNA expressions of NOX1, 2, and 4 in aortas (Figure 5E) (Supplemental Figure S3C,D). AngII increased the expression of NOX2 in aortas from WT mice, but this was blunted in those from SOD1^−/−^ mice (Figure 5E). This would be related to aortic ROSs production by AngII, which was attenuated in SOD1^−/−^ mice.

### 3.6. Rat VSMCs Incubated with AngII

**Figure 6:**
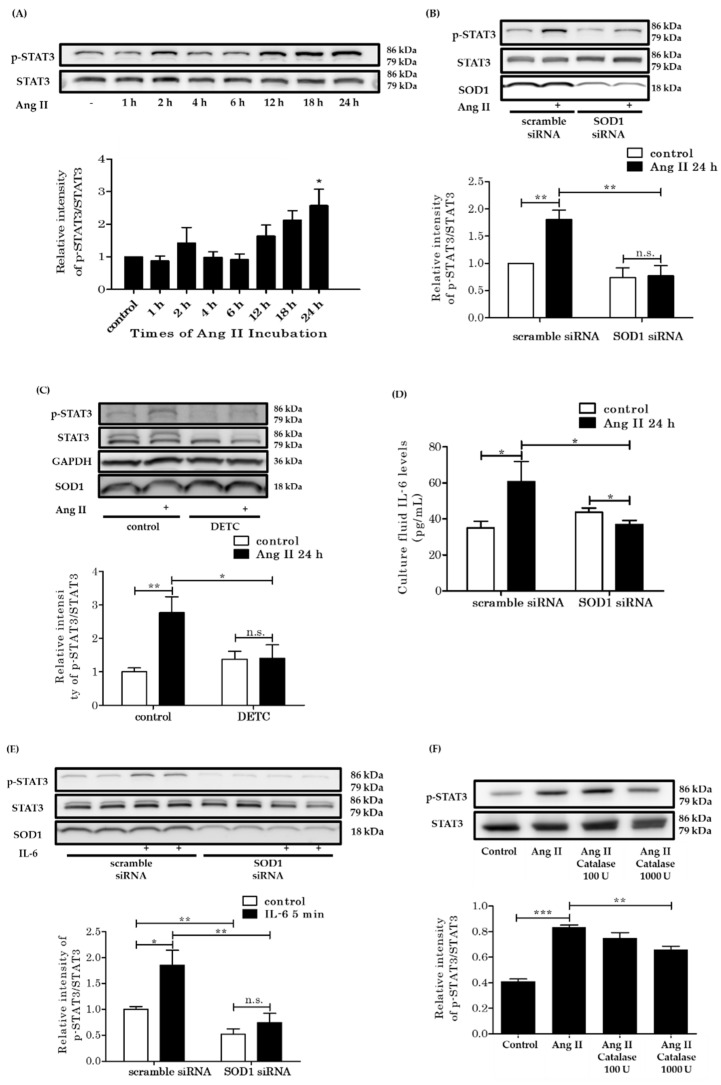
Rat vascular smooth muscle cells (VSMCs) incubated with AngII. (**A**) Time course of STAT3 phosphorylation of VSMCs due to AngII. VSMCs were stimulated with 100 nM AngII for the indicated times. The top panels are representative immunoblots for Ang II-induced phosphorylation of STAT3. The bottom panels represent averaged data quantified by densitometry of immunoblots, expressed as fold increases in phosphorylation, in which the phosphorylation for the control was defined as 1.0 (*n* = 4 per group. * *p* < 0.05) (**B**) The expression of phosphorylated STAT3 of VSMCs induced with AngII. The expression of SOD1 was downregulated with SOD1 siRNA (6 nM). The bottom panels represent quantification of densitometry, expressed as fold increases in phosphorylation, in which the phosphorylation for “scramble siRNA control’’ was defined as 1.0 (*n* = 7 per group. * *p* < 0.05 ** *p* < 0.01 n.s.: not significant). (**C**) The SOD1 activity was pharmacologically inhibited by 0.1 mM DETC (copper-chelating compound). DETC was added to VSMC culture fluid 4 h before AngII. The bottom panels represent averaged data quantified by densitometry of immunoblots (*n* = 9 per group. * *p* < 0.05 ** *p* < 0.01 n.s.: not significant). (**D**) The IL-6 levels in the culture fluid were analyzed by the ELISA test (*n* = 12–15 per group. * *p* < 0.05). (**E**) The expression of phosphorylated STAT3 of VSMCs induced with IL-5. The expression of SOD1 was downregulated with SOD1 siRNA (6 nM). The bottom panels represent quantification of densitometry, expressed as fold increases in phosphorylation, in which the phosphorylation for “scramble siRNA control’’ was defined as 1.0 (*n* = 7 per group. * *p* < 0.05 ** *p* < 0.01 n.s.: not significant) (**F**) The top panels are representative immunoblots of Ang II-induced phosphorylation of STAT3 in the presence and absence of PEG-catalase (100–1000 U). The bottom panels represent averaged data quantified by densitometry of immunoblots (*n* = 3 per group. ** *p* < 0.01 *** *p* < 0.001). Error bars represent SEM. Ang II: angiotensin II; STAT3: signal transducer and activator of transcription 3; GAPDH: glyceraldehyde-3-phosphate dehydrogenase; SOD1: superoxide dismutase 1; DETC: diethyldithiocarbamate; AngII: angiotensin II; PEG-catalase: polyethylene glycol-conjugated catalase.

In the present AngII infusion model, STAT3 was phosphorylated in the aorta in vivo. So, we next examined the phosphorylation of STAT3 by AngII in vitro. We cultured rat VSMCs and incubated them with 100 nM AngII. STAT3 was not activated in a short period of time but its phosphorylation was significantly increased after 24 h (Figure 6A). This meant that phosphorylation of STAT3 due to AngII required time. We down-regulated SOD1 expression in rat VSMCs using SOD1 siRNA (6 nM). In the control VSMCs, AngII had induced STAT3 phosphorylation at 24 h, though this response was blunted in VSMCs treated with SOD1 siRNA (Figure 6B). VSMCs were also incubated with 0.1 mM diethyldithiocarbamate (DETC) to inhibit the activity of SOD1 pharmacologically. DETC blunted the phosphorylation of STAT3 in incubation with AngII for 24 h (Figure 6C).

The effect of AngII has been observed to be polymorphic, with some tyrosine-phosphorylated proteins being phosphorylated more rapidly by AngII [33]. On the other hand, it took 24 h to phosphorylate STAT3 by AngII. The phosphorylation of STAT3 was blunted by SOD1 downregulation and SOD1 inhibitor, suggesting that the phosphorylation of STAT3 by AngII involves the generation of intermediates, which may be affected by SOD1. The in vivo study suggested that IL-6 was one of the intermediates. IL-6 levels in the culture fluid were increased by AngII in control VSMCs, but not in SOD1 siRNA-treated VSMCs (Figure 6D). In other words, the secretion of IL-6 by AngII was decreased in VSMCs with reduced expression of SOD1.

Next, we directly administered IL-6 (1 ng/mL) to the VSMCs and examined the phosphorylation of STAT3 by western blot analysis. With down-regulation of SOD1, STAT3 phosphorylation due to incubation with IL-6 was blunted (Figure 6E). These results suggest that SOD1 is involved in IL-6 secretion from VSMCs and phosphorylation of STAT3 by IL-6 in the presence of AngII.

SOD1 produces H_2_O_2_ and O_2_ from two O_2_^·−^ in the cytoplasm. The effect of SOD1 observed above can be considered as a result of the change in the cytoplasmic H_2_O_2_ concentration. Catalase is an enzyme that metabolizes H_2_O_2_, and since H_2_O_2_ can freely pass through the cell membrane, the addition of catalase extracellularly decreases the intracellular H_2_O_2_ level. Therefore, we administered catalase into the culture medium 24 h before AngII administration and measured the expression of phosphorylated STAT3 in VSMCs. The administration of catalase (1000 U) attenuated the phosphorylation of STAT3 (Figure 6F).

## 4. Discussion

In this study, we examined the role of SOD1 in vascular physiology and pathophysiology using a well-characterized SOD1 knockout mouse, in particular focusing on aortic hypertrophy and inflammation. We found that knocking out SOD1 alone did not cause hypertension or aortic pathology, going against the common belief that SOD1 has an antioxidant role. Moreover, in the Ang II infusion model, deletion of SOD1 reduced vascular pathology despite a similar increase in blood pressure as in WT. Serum IL-6 levels were increased to a similar extent but the up-regulation in aortic expression of IL-6 mRNA under AngII infusion was markedly blunted in SOD1^−/−^ mice. The phosphorylation of STAT3, which involves downstream signaling of IL-6, was also blunted in SOD1^−/−^ mice. Real time-PCR indicated that the AngII-induced up-regulation of NOX2 and the inflammation-related cytokines (IL-1β, IL-6, and MCP-1) was decreased in the aorta from SOD1^−/−^ mice. In VSMCs cultured with AngII, inhibition of SOD1 by siRNA or inhibitor suppressed IL-6 secretion, reduced the effect of IL-6 itself, and suppressed the inflammatory STAT3 cascade. PEG-catalase also inhibited STAT3 phosphorylation in the presence of AngII.

### 4.1. SOD1 Changed ROS Levels

SOD1 is mainly localized in the cytosol and mitochondrial intermembrane spaces. We postulated that intracellular O_2_^·−^ would be upregulated and H_2_O_2_ would be downregulated in SOD1^−/−^ mice aortas both under physiological conditions and under AngII infusion. However, under physiological conditions, cytosolic O_2_^·−^ levels detected by DHE staining and H_2_O_2_ levels estimated from the GSSG/GSH ratio were not different in both mice. On the other hand, it has been reported that O_2_^·−^ levels detected by lucigenin and DHE staining in SOD1^−/−^ mice are higher than in WT mice under physiological conditions [24]. One possible reason for this discrepancy is that we used relatively young mice. In this regard, it has been reported that O_2_^·−^ levels in blood vessels are high in old subjects [41], and O_2_^·−^ is mainly produced in mitochondria, it is possible that the O_2_^·−^ produced in the young cells was rapidly degraded by SOD2, and there was less O_2_^·−^ leaking into the cytoplasm itself. In AngII-treated mice, the GSSG/GSH ratio in the aorta was increased and DHE staining was enhanced in both mice, but the differences were not significant. On the other hand, in RBCs that lack SOD2, the GSSG/GSH ratio, i.e., the increase in H_2_O_2_, was attenuated in SOD1^−/−^ mice by Ang II treatment. This suggests that the presence of SOD2 compensated for the effect of SOD1 deficiency in the cells of the aortas as a whole, and that the change in ROS in the whole vessel might be masked. Since some reactive thiols on proteins (such as small GTPase Ras, phosphatases and so on) are susceptible to H_2_O_2_ (hydroxyl radical) [42,43,44]. Thus, relatively small changes in H_2_O_2_ which may not be detected by GSSG/GSH in cytosol, could have significant effects on local signaling molecules related to hypertrophy in VSMCs.

Real-time PCR showed that NOX2 was not upregulated in the presence of AngII in SOD1^−/−^ mice, suggesting that aortic local O_2_^·−^ production was also reduced by SOD1 deficiency. However, ROSs detected by DHE staining was no difference between WT and SOD1^−/−^ mice (Supplemental Figure S2). Thus, the contribution of NOX2 to overall aortic ROS production may be limited. One of the mechanisms for the reduced expression of NOX2 in SOD1^−/−^ may be due to the molecular interaction between NOX2 and STAT3 [45]. Extracellular H_2_O_2_ levels in serum were detected using d-ROMs. There were similar increases in serum d-ROM levels due to AngII infusion in SOD1^−/−^ and WT mice, suggesting that SOD3 catalyzed O_2_^·−^ to an equal extent.

### 4.2. Hypertensive Response

AngII behaves as a vasopressor hormone via G protein-mediated pathways [4]. In addition, AngII post-translationally activates multiple NOX isoforms (NOX1, NOX2, and NOX5), and in the later phase, it also up-regulates the expressions of NOX enzymes [46]. By means of these mechanisms, AngII produces O_2_^·−^, which decreases the bioavailability of NO and induces hypertension. O_2_^·−^ is rapidly catalyzed to H_2_O_2_ by SOD [14]. Furthermore, cytosol H_2_O_2_ has been assumed to act as a vasodilator known as an endothelium-derived hyperpolarizing factor (EDHF) [47].

The role of SOD in the hypertensive response may vary with its individual isoforms as well as their localization. For example, in extracellular SOD (SOD3)-deficient mice, the AngII-induced hypertensive response was enhanced [48] and brain SOD3 was especially important in blood pressure modulation [49,50]. In addition, adenovirus-mediated gene transfer of SOD3 decreased blood pressure and improved endothelium-dependent relaxation in the spontaneous hypertensive rat [51]. The findings of these studies clearly indicated that SOD3 reduces the hypertensive response and that the elimination of extracellular O_2_^·−^ by SOD3 is important to the bioavailability of NO and blood pressure.

SOD1 is localized in the cytosol and mitochondrial intermembrane spaces and its role in the pressor response is complex and controversial; one study suggested that SOD1 had no remarkable role in blood pressure in the presence of AngII [20] and others suggested that it had anti-hypertensive roles [21,22,23]. There was also a discrepancy in basal blood pressures [20,21,22,23]. These previous inconsistent results suggest that the modulation of blood pressure by SOD1 might depend on age, methods of inducing hypertension, and study settings. One difference, in conclusion, may have resulted from employing SOD1 transgenic or SOD1^−/−^ mice. The Ang II dose was varied depending on study design (0.4–5mg/kg/day 7–14days) [23,52,53,54,55]. In the present study, we aimed to evaluate aortic remodeling and used a relatively high concentration of AngII (3.2 mg/kg/day) in SOD1^−/−^ mice as in previous studies [22] but no difference in O_2_^·−^ level was observed as described above. Our results showed there was no difference in basal blood pressure and a similar increase was observed in the presence of AngII for seven days.

Some studies used radiotelemetry to measure blood pressure, however, we used the noninvasive tail-cuff method as previously [20,22,26]. To reduce the stress associated with the BP measurements and variations, mice were introduced into the plastic restrainer for about 30 min from one week before the main experiment and 10 times averages were calculated at each determination. The non-invasive method to measure blood pressure has been reported to correlate well with intra-arterial measurements [56].

### 4.3. Aortic Remodeling

Some studies have shown that SOD1^−/−^ mice exhibit baseline vascular hypertrophy in cerebral arterioles and renal afferent arterioles [21,23]. We did not investigate arterioles but observed that the baseline aortic thickness of SOD1^−/−^ mice was similar to that of WT mice. In our study, SOD1^−/−^ mice revealed less aortic hypertrophy under AngII infusion in spite of similar pressor responses. Wang et al. reported that in mice overexpressing human SOD1, the aortic wall thickened to the same extent as in WT mice, even though the Ang II-induced pressure increase response was attenuated [22]. From this study and ours, we found that the presence of SOD1 promoted aortic remodeling in response to AngII administration, and the degree of remodeling was at least partially independent of the AngII-induced pressure-raising response.

In vitro studies have shown that ROS induced by AngII plays an important role in VSMC hypertrophy [4,9,10,17,18,57]. Many studies have also shown that intracellular H_2_O_2_ production is an important mediator of protein synthesis in VSMCs in vitro [15,16]. In an in vivo study, NOX promoted aortic hypertrophy in a hypertensive model infused with AngII, while medial hypertrophy was reduced in NOX2-deficient mice [26]. Zhang et al. reported that in VSMCs specific catalase overexpression mice, Ang II-induced aortic hypertrophy was attenuated in spite of a similar hypertensive response with control mice [58]. These studies are consistent with our hypothesis that O_2_^·−^ produced by Ang II is metabolized to cytosolic H_2_O_2_ in the presence of SOD1, and that this H_2_O_2_ causes VSMC hypertrophy, leading to aortic thickening in vivo.

AngII is a multifunction hormone, and activates a complex series of intracellular signaling pathways for VSMC growth and proliferation [33], including extracellular signal-regulated kinase 1/2 (ERK1/2), p38 mitogen-activated protein (MAP) kinases, Akt, c-jun N-terminal kinases, Rho kinases, Ras and Janus kinase2/Signal transducer and activator of transcription 3 (JAK2/STAT3) pathways [4,59,60]. Mitogen-activated protein kinases, including ERK1/2, have been implicated in VSMC differentiation, migration, and proliferation. Moreover, the cell survival protein kinase, Akt/protein kinase B, has been seen to be involved in AngII induced protein synthesis and AngII is known to activate Akt via the production of H_2_O_2_ from NOX in vitro [34,37]. However, in our in vivo study, the phosphorylation of ERK1/2 and Akt was not enhanced by AngII-infusion at seven days. Although we cannot rule out the possibility that these kinases are transiently activated at earlier stages, we did not think that they played an important role in our in vivo aortic remodeling model. Therefore, we examined other AngII-related kinases that are constantly suppressed by SOD1 deletion after seven days of AngII treatment.

AngII promotes the inflammatory JAK/STAT pathway [61]. In other studies, the JAK/STAT pathway responded to intracellular H_2_O_2_ generation [53,62], and AngII-induced activation of the JAK2/STAT3 pathway required NOX systems [38]. Therefore, we investigated whether the JAK2/STAT3 pathway is involved in the aorta used in the study. AngII-infusion had enhanced the phosphorylation of STAT3 in the aortas of WT mice at seven days, and interestingly, this was blunted in SOD1^−/−^ mice. Marrero et al. reported that activation of STAT3 by AngII was observed after 60 min [35] and therefore, AngII-induced STAT3 activation might be mediated by autocrine/paracrine mechanisms. We examined mechanisms further in cultured VSMCs. Consistent with their study, we could not detect enhanced phosphorylation of STAT3 in VSMCs incubated with AngII within a short period, but phosphorylation was enhanced after 24 h. The slow phosphorylation of STAT3 was blunted by genetic and pharmacological inhibition of SOD1 and by PEG-catalase. These findings suggested that AngII activates STAT3 in an autocrine/paracrine manner and that the response requires H_2_O_2_ generation by SOD1, leading to aortic hypertrophy. Cheng JF et al. reported that AngII induces proliferation of aortic VSMCs through activation of the JAK2/STAT3 signaling pathway [63], which is consistent with our findings.

### 4.4. Inflammation and IL-6 Production

**Figure 7:**
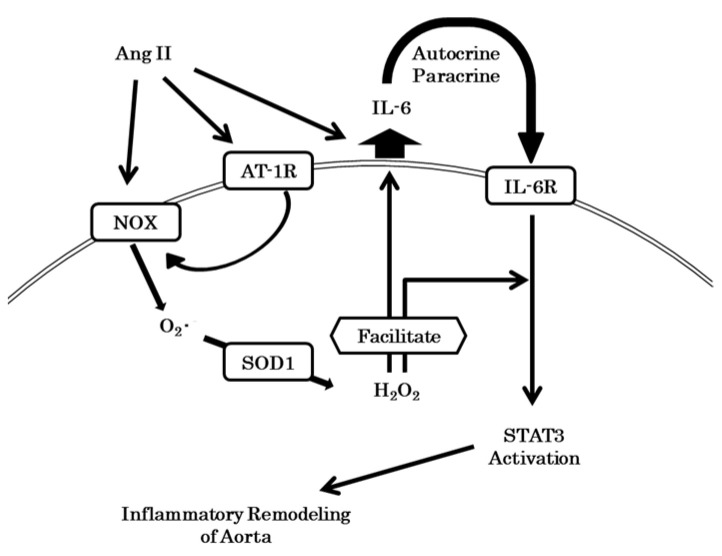
The cytosolic H_2_O_2_ produced by SOD1 facilitated IL-6 synthesis in VSMCs and its secretion from them, which had been induced by AngII. H_2_O_2_ facilitated the activation of STAT3 by IL-6. These mechanisms were responsible for the inflammatory remodeling of aortas. AT-1R: angiotensin II receptor 1; NOX: NADPH oxidase.

Many cytokines and growth factors activate the JAK2/STAT3 pathway, and IL-6 is representative of such cytokines. Previous studies have indicated that AngII induced IL-6 synthesis in VSMCs as well as its release from them, and these processes required ROS generated by NOX systems [38,39,40]. We considered that IL-6 is one of the intermediate substances that activates STAT3 under Ang II administration in the present study. In the presence of AngII, elevations of serum IL-6 levels were similar in both WT mice and SOD1^−/−^ mice, whereas the up-regulation of aortic IL-6 RNA expression was blunted in SOD1^−/−^ mice. Under AngII infusion, the aortic expressions of other cytokines, such as MCP-1 and IL-1β were also increased but were blunted in SOD1^−/−^ mice. In a study using cultured VSMCs, AngII had increased IL-6 release into the culture fluid in control VSMCs at 24 h, however, the release of IL-6 was reduced in VSMCs in which SOD1 was down-regulated. The rapid phosphorylation of STAT3 due to IL-6 seen at 5 min was also blunted in VSMCs with down-regulated SOD1. These results indicate that SOD1 was necessary to induce the synthesis and secretion of IL-6 and that it also enhanced the phosphorylation of STAT3 (Figure 7). We considered that SOD1 in aortic smooth muscle promoted inflammatory remodeling under AngII infusion via cytosolic H_2_O_2_ production and modulation of IL-6/STAT3 signaling.

In previous studies using IL-6 deficient mice, aortic O_2_^·−^ production induced by AngII was blunted [64] and AngII-induced retinal vascular hypertrophy/remodeling was suppressed [52]. AngII induced the production of IL-6, which mediated remodeling/hypertrophy in vascular cells [54,55] and cardiomyocytes [65] in an autocrine/paracrine manner. Together, our results and these previous findings indicate that there would be close interactions between AngII induced ROS generation and inflammation-related cytokines, especially IL-6. It is known that innate and acquired immune cells are involved in one mechanism of hypertension [66]. In hypertensive blood vessels, immune cells not only produce ROS, but also promote ROS production from vascular cells by cytokines produced, which plays a role in vascular damage in hypertension [67]. It is possible that IL-6 secretion and ROS in immune cells are also regulated by SOD1 and may affect blood pressure and aortic remodeling.

As H_2_O_2_ has been reported to modulate many transcription factors, kinases, small GTPases, and phosphatases, these molecules might be involved in the modulation of IL-6 production and STAT3 activation due to IL-6. For example, transcription of IL-6 depends on NF-kappa B activation [68] and H_2_O_2_ can activate NF-kappa B although the mechanism has not been completely clarified [69,70]. H_2_O_2_ attacks the cysteine thiol of SH2 containing phosphatase (SHP), which is in the active center, and inhibits SHP activity [71,72,73]. The decreased cytosolic H_2_O_2_ production through deletion of SOD1 in VSMCs might activate SHP and dephosphorylate and inhibit the JAK/STAT pathway. Further studies will be required to clarify precise mechanisms for IL-6 production and STAT3 activation due to AngII in a SOD1-dependent manner.

## 5. Conclusions

In conclusion, by comparing SOD1^−/−^ with WT mice, we found that SOD1 function in the vasculature is limited under physiological conditions, but in the presence of Ang II, it causes aortic hypertrophy independent of the pressure increase response. Because of the presence of mitochondria in vascular cells, it was inferred that SOD2 compensated for SOD1 deficiency, and no changes in net ROS were observed in the aorta as a whole. However, the elevation of cytoplasmic local H_2_O_2_ produced by SOD1 was thought to cause aortic hypertrophy by targeting signaling molecules such as Ras and phosphatases [42,43,44] via secretion of IL-6 and activation of inflammatory STAT3 in VSMCs. These results suggest that SOD1 is involved in aortic remodeling in Ang II-induced hypertension via regulation of the inflammatory pathway IL-6/STAT3 axis in VSMCs.

## Figures and Tables

**Table 1:** Primer pairs for quantitative real-time PCR.

Gene	Sequence		Product Suze (bp)
Il6 (IL-6)	Forward:	5′-ACAACCACGGCCTTCCCTACTT-3′	129
	Reverse:	5′-CACGATTTCCCAGAGAACATGTG-3′	
Ccl2 (MCP-1)	Forward:	5′-CCACTCACCTGCTGCTACTCAT-3′	76
	Reverse:	5′-TGGTGATCCTCTTGTAGCTCTCC-3′	
Il1b (IL-1beta)	Forward:	5′-GCTGCTTCCAAACCTTTGAC-3′	118
	Reverse:	5′-TTCTCCACAGCCACAATGAG-3′	
Nox1	Forward:	5′-CTACAGAAGAAGCCAACAGGCCAT-3′	117
	Reverse:	5′-ACTGTCATGTTTGGAGACTGGATG-3′	
Cybb (Nox2)	Forward:	5′-CCCTTTGGTACAGCCAGTGAAGAT-3′	67
	Reverse:	5′-CAATCCCGGCTCCCACTAACATCA-3′	
Nox4	Forward:	5′-GGATCACAGAAGGTCCCTAGCAG-3′	113
	Reverse:	5′-GCGGCTACATGCACACCTGAGAA-3′	
Rn18s (18S rRNA)	Forward:	5′-TTCCGATAACGAACGAGACTCT-3′	99
	Reverse:	5′-TGGCTGAACGCCACTTGTC-3′	

## Data Availability

The data presented in this study are available from the corresponding author on reasonable request.

## Supplementary Materials

The following are available online at https://www.mdpi.com/2076-3921/10/3/471/s1, Figure S1: Microscopic appearance of aortic rings, Figure S2: Appearance and quantitative analysis of DHE staining of the aorta, Figure S3: Serum TNF-α concentration and TNF-α, NOX1, and NOX4 mRNA expression in aortas.
