# Supplementary material for: Deletion of Superoxide Dismutase 1 Blunted Inflammatory Aortic Remodeling in Hypertensive Mice under Angiotensin II Infusion
Source: Antioxidants (Basel). 2021 Mar 16;10(3):471. doi: 10.3390/antiox10030471 (PMC8002308; doi:10.3390/antiox10030471)
Supplement: Supplementary file 1 [file antioxidants-10-00471-s001.zip › supplement/Figure S1.docx]

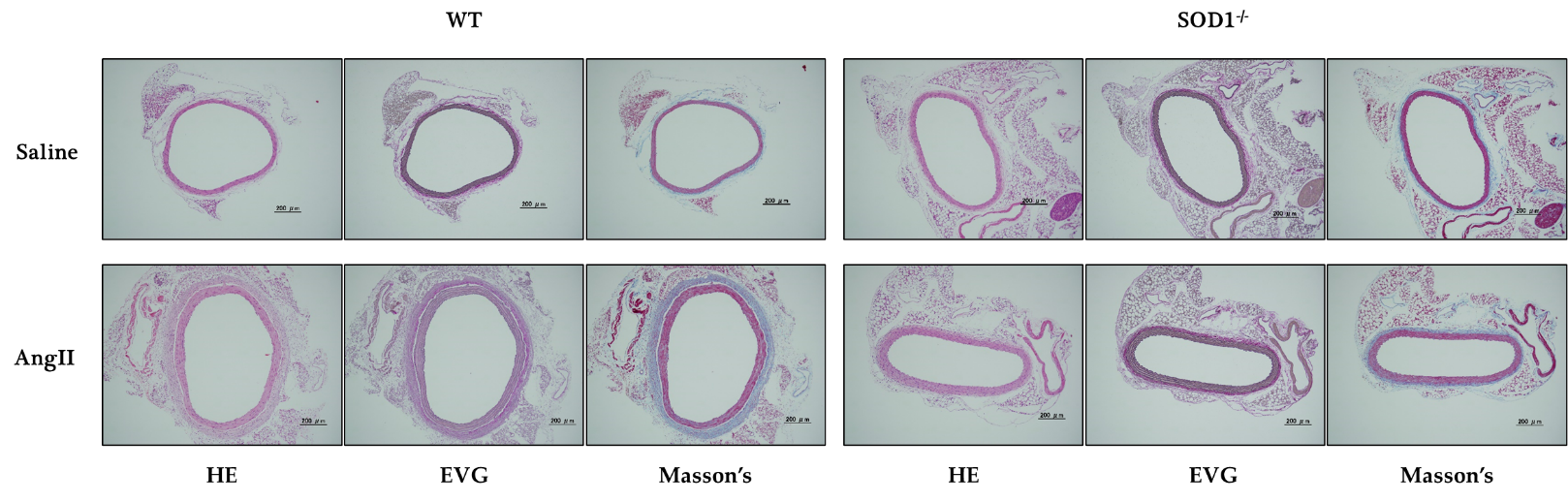


**Figure S1.** Microscopic appearance of aortic rings. HE, EVG, and Masson’s staining of aortas. WT mice with saline (upper left), SOD1^−/−^ mice with saline (upper right), WT mice with AngII (lower left), SOD1^−/−^ mice with AngII (lower right). (Scale bar = 200 μm.) HE: Hematoxylin-Eosin Stain; EVG: Elastica van Gieson stain; Masson’s: Masson's trichrome stain.
