# Supplementary material for: Deletion of Superoxide Dismutase 1 Blunted Inflammatory Aortic Remodeling in Hypertensive Mice under Angiotensin II Infusion
Source: Antioxidants (Basel). 2021 Mar 16;10(3):471. doi: 10.3390/antiox10030471 (PMC8002308; doi:10.3390/antiox10030471)
Supplement: Supplementary file 1 [file antioxidants-10-00471-s001.zip › supplement/Figure S2.docx]

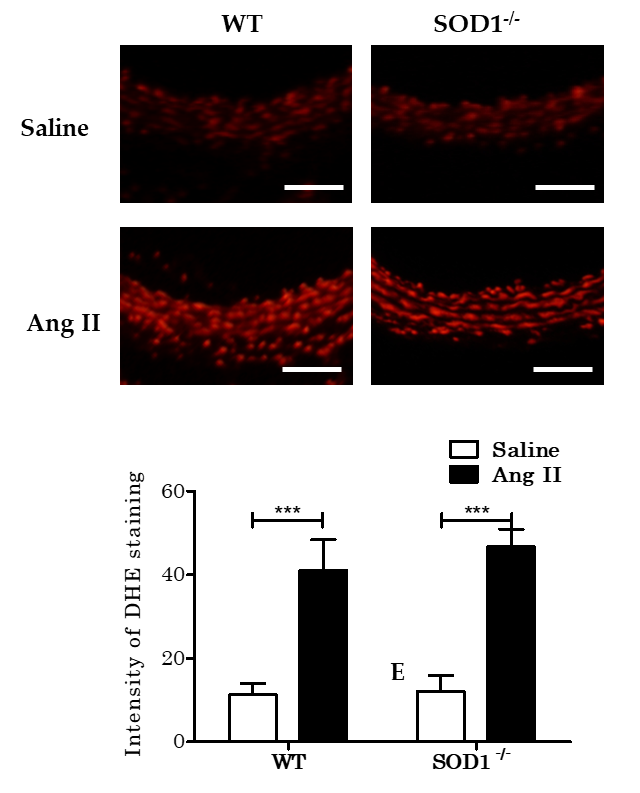


**Figure S2.** Appearance and quantitative analysis of DHE staining of the aorta. Upper: Vascular O_2_･^−^ levels were assessed with the DHE stain. Lower: Summarized data for the intensity of DHE staining (*n* = 7~8 per group. ****p* < 0.001). Error bars represent SEM. ROS: reactive oxygen species; WT: wild type; SOD1^−/−^: superoxide dismutase 1 deficient; Ang II: angiotensin II; DHE: Dihydroethidium.
