# Supplementary material for: Deletion of Superoxide Dismutase 1 Blunted Inflammatory Aortic Remodeling in Hypertensive Mice under Angiotensin II Infusion
Source: Antioxidants (Basel). 2021 Mar 16;10(3):471. doi: 10.3390/antiox10030471 (PMC8002308; doi:10.3390/antiox10030471)
Supplement: Supplementary file 1 [file antioxidants-10-00471-s001.zip › supplement/Figure S3.docx]

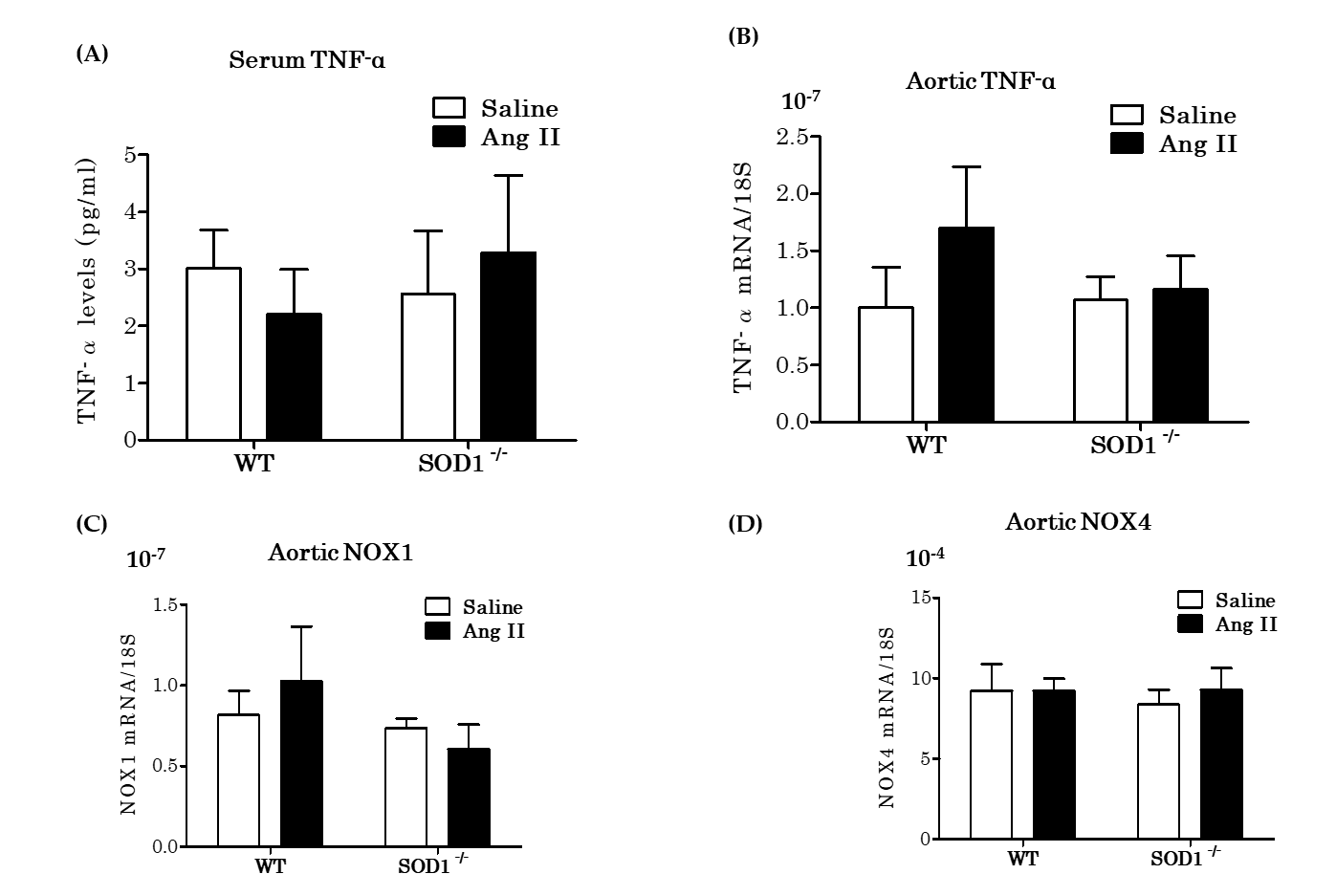


Figure S3.

Serum TNF-α concentration and TNF-α, NOX1, and NOX4 mRNA expression in aortas**.** (A) Serum TNF-α levels were analyzed by the ELISA test (n=9~10 per group). (B~D) Local aortic TNF-α, NOX1, andNOX4 levels were detected as mRNA expressions, which were compensated with an internal control: 18S (n=4~8 per group). Error bars represent SEM. WT: wild type; SOD1^-/-^: superoxide dismutase 1 deficient; Ang II: angiotensin II; TNF: tumor necrosis factor; NOX: NADPH Oxidase.
